# Spray Drying Encapsulation of *Pediococcus acidilactici* at Different Inlet Air Temperatures and Wall Material Ratios

**DOI:** 10.3390/foods12010165

**Published:** 2022-12-28

**Authors:** Gabriella Devina Tirta, Leon Martin, Mario Donald Bani, Katherine Kho, Ihsan Tria Pramanda, Liew Phing Pui, Yu Hsuan How, Crystale Siew Ying Lim, Putu Virgina Partha Devanthi

**Affiliations:** 1 Department of Biotechnology, School of Life Sciences, Indonesia International Institute for Life Sciences, Pulomas Barat Kavling 88, Jakarta 13210, Indonesia; 2 Department of Food Science and Nutrition, Faculty of Applied Sciences, UCSI University, Jalan Menara Gading, UCSI Heights, Cheras, Kuala Lumpur 56000, Malaysia; 3 Department of Biotechnology, Faculty of Applied Sciences, UCSI University, Jalan Menara Gading, UCSI Heights, Cheras, Kuala Lumpur 56000, Malaysia

**Keywords:** probiotic, encapsulation, spray drying, *Pediococcus acidilactici*, whey protein isolate, gum arabic, inlet temperature

## Abstract

*Pediococcus acidilactici* has gained research and commercial interest due to its outstanding probiotic properties, yet its survival during storage and consumption requires improvement. This study aims to enhance *P. acidilactici* survival using spray drying encapsulation. Different inlet air temperatures (120 °C, 150 °C, and 170 °C) and whey protein isolate (WPI):gum arabic (GA) ratios (1:1, 3:1, 1:3) were tested. Cell viability was significantly (*p* < 0.05) affected by the inlet temperature but not the WPI:GA ratio. Increasing the inlet temperature to 170 °C significantly decreased *P. acidilactici* viability by 1.36 log cycles, from 8.61 log CFU/g to 7.25 log CFU/g. The inlet temperature of 150 °C resulted in a powder yield (63.12%) higher than at 120 °C (58.97%), as well as significantly (*p* < 0.05) lower moisture content (5.71%) and water activity (a_w_ 0.21). Viable cell counts in all encapsulated *P. acidilactici* were maintained at 5.24–6.75 log CFU/g after gastrointestinal tract (GIT) simulation, with WPI:GA of 3:1 and inlet temperature 150 °C having the smallest log reduction (0.3 log cycles). All samples containing different WPI:GA ratios maintained sufficient viability (>7 log CFU/g) during the first three weeks of storage at 25 °C. These results could provide insights for further developing *P. acidilactici* as commercial probiotic products.

## 1. Introduction

Probiotics are living microorganisms that provide health benefits to their host (or consumer) by maintaining the gut microbiome balance. Their benefits may include intestinal health improvement, immune response enhancement, and better serum cholesterol control [1]. Nowadays, probiotics are commercially available as dietary supplements (e.g., probiotic capsules and tablets [2]) and functional foods (e.g., yogurt [3], cheese [4], cereal [5], chocolate [6], and fruit juice [7]). However, to deliver their health benefits, a sufficient cell concentration must be administered in the food products. In general, it is recommended to incorporate 6–7 log probiotic cells per mL or gram product [1,8,9,10], and a total of 9–10 log probiotic cells should be consumed daily [1,11].

*Pediococcus acidilactici* are homofermentative gram-positive lactic acid bacteria known to have a great capacity to survive the harsh environment in the animal and human digestive systems (e.g., acidic pH, pepsin, and bile salt), enabling gut colonization [12,13]. Over the past decades, it has considerably gained research interest due to its potent probiotic characteristics, such as good antimicrobial activity [14,15], good adherence to intestinal cells [14,15], and the capability to produce useful metabolites such as bacteriocin and gamma-aminobutyric acid [16,17]. In addition, it has wide applications in the food industry, ranging from fish feed supplementation [18,19], starter culture for traditional sausage production [20], orange juice supplementation [21,22], and bio-preservative agents in food products [23]. However, during processing, storage, and consumption in the gastrointestinal tract (GIT), the probiotics are exposed to several environmental stresses, including heat, desiccation, and low pH, which reduce their viability [21,24,25,26,27]. The probiotics’ survival against such environmental stress could be improved by encapsulation [24,25].

Spray drying is a commonly used encapsulation technique [28,29,30], as it is simple, fast, cost-effective, and scalable compared to other drying encapsulation methods [31,32]. In spray drying encapsulation, probiotics are added to the encapsulation material solution, known as feed solution, then atomized into small particles, exposed to hot air (150 °C to 250 °C), and transformed into powders [33,34]. The resulting powder will contain probiotics encapsulated within the coating matrix that protects the cells from upcoming environmental stress [35]. However, spray drying exposes the probiotics to heat and desiccation stress, which could reduce the viable cell number [31,36,37]. Hence, selecting appropriate parameters used in spray drying is important to maintain a high encapsulation efficiency [38,39,40].

One of the critical parameters in spray drying is inlet air temperature, which refers to the pre-heated drying air entering the drying chamber [41]. Choosing a suitable inlet air temperature is critical in obtaining good quality powder, such as low moisture content and water activity, which is required to prevent microbial growth or contamination and maintain product stability during storage [38,40]. Studies by Flores et al. [39] and Ortega and Vandeker [40] have shown that high inlet air temperature is favourable as it results in low moisture content and water activity. However, such high temperature also induces viable cell loss as it gives relatively high stress that could damage the cell wall, DNA, and RNA and disrupt their metabolic activity [8,40,42].

Besides the inlet temperature, selecting an appropriate encapsulation material is also crucial to obtain a powder with high viable cell concentration. No single biopolymer can provide all the ideal criteria for encapsulation materials (e.g., edible, low-cost, idle in nature, and good physicochemical properties) [43]. Hence, two or three materials are often used to obtain synergic properties in maintaining a high viable probiotic cell count [44]. Whey protein isolate (WPI) and gum arabic (GA) have been shown to have good properties as spray drying encapsulation materials, as they are able to construct physically strong and stable matrices [45]. Furthermore, their interactions demonstrate excellent interfacial activity and emulsifying properties that can protect the probiotic cells during spray drying [46]. Another study demonstrates that WPI, in combination with GA, exhibited the highest probiotic survival during GIT simulation compared to WPI combined with other materials, such as locust bean gum and maltodextrin [47]. Moreover, using response surface methodology, the use of WPI and GA was predicted to encapsulate *Lactobacillus acidophilus* with a high encapsulation efficiency of 93.95% [44].

As of now, the effect of inlet air temperature and the use of WPI and GA as spray drying encapsulation material for *P. acidilactici* has never been studied. Hence, this study investigated the effect of varying inlet temperature (120 °C, 150 °C and 170 °C) and WPI:GA ratio (1:1, 3:1, and 1:3) on *P. acidilactici* survivability during spray drying, storage, and GIT simulation. Additionally, the production yield and physicochemical properties of all samples were analysed, including moisture content, water activity, Fourier-transform infrared spectroscopy (FT-IR), and scanning electron microscopy (SEM).

## 2. Materials and Methods

### 2.1. Materials and Culture

Encapsulation materials were food grade, and the other materials for survival and physicochemical analysis were analytical grades. Whey protein isolate 90 (WPI) (JFO store, Jakarta, Indonesia) and gum arabic (GA) (ALMA Chemical, Demak, Indonesia) were purchased using local e-commerce in Indonesia. Microbiological growth media used were de Man-Rogosa-Sharpe (MRS) broth (MERCK, Darmstadt, Germany) and MRS agar (MERCK, Darmstadt, Germany). For GIT simulation, the MRS broth was supplemented with glucose (MERCK, Darmstadt, Germany), KH_2_PO_4_ (MERCK, Darmstadt, Germany), CaCl_2_ (MERCK, Darmstadt, Germany), and KCl (MERCK, Darmstadt, Germany). The rehydration media for spray-dried probiotic and an additional supplement to gastrointestinal simulation media was NaCl (Himedia, Mumbai, India). The pH of gastrointestinal simulation media was maintained by adding HCl (MERCK, Darmstadt, Germany) and NaOH (ROFA, Bandung, Indonesia). *P. acidilactici* culture was obtained from Universitas Gadjah Mada (UGM), Food and Nutrition Culture Collection. The bacterial identity was confirmed through gram-staining and 16S rRNA sequencing.

### 2.2. Culture Preparation for Spray Drying

*P. acidilactici* from MRS agar was subcultured twice in fresh 50 mL MRS broth, followed by incubation at 30 °C for 18 h until it entered the late log phase. Then, 50 mL culture (OD_600_ 0.7 equal to 7–8 log CFU/mL) was harvested and washed twice with 25 mL 0.9% NaCl solution. Cell harvest and wash were done by centrifugation at 2438× *g*, 25 °C, for 15 min. Lastly, the culture was concentrated by removing the supernatant, and the pellet was resuspended in 10 mL 0.9% NaCl solution to get a higher cell concentration (9–10 log CFU/mL).

### 2.3. Viable Cell Counting

The viable cell counting was performed using the Miles and Misra method with adjustments [48]. First, 100 µL culture or 1 g of spray-dried sample was transferred to 900 µL or 9 mL 0.9% NaCl solution, respectively, followed by serial dilution. Then three drops of 10 µL culture from each dilution were dropped onto the MRS agar and allowed to set. The agar plates were incubated at 30 °C for 36–48 h, and the number of colonies was calculated.

### 2.4. Spray Drying

Feed solution containing probiotic culture (1% *v*/*v*) and 20% *w*/*v* WPI-GA was prepared to be subjected to spray drying. The effect of inlet temperature was investigated by keeping the WPI-GA ratio fixed at 1:1, while varying the inlet temperature at 120 °C, 150 °C and 170 °C. To evaluate the effect of WPI:GA ratio, the inlet temperature was fixed at 150 °C, while WPI:GA ratio was varied to 1:3, 1:1, and 3:1.

After the culture was prepared (described in Section 2.2), feed solutions containing encapsulation material were prepared. A total of 200 g WPI and GA were dissolved in 1 L mineral water and the solution was homogenized using a hand blender (Bamix Deluxe hand blender, Mettlen, Switzerland) for 5 min at maximum speed. Once homogenized, the 10 mL culture prepared in Section 2.2 was added and the feed solution was homogenized for another 2 min. The spray drying was carried out using a pilot-scale spray dryer (LPG 5, Changzhou Huaihai Drying Equipment Co., Ltd., Changzhou, China). The solutions were fed into the chamber through a peristaltic pump at a constant flow rate of 15 rpm/min. Other parameters were fan speed at 45 Hz, atomisation at 250 Hz, and air hammer within 1 s every 20 s. Spray-dried samples were cooled down and further stored in a Ziplock plastic bag. The Ziplock bag was kept inside an aluminum bag, added with silica gel, and sealed with a heat sealer until further analysis.

### 2.5. Viability Loss

The viability loss was defined as a log reduction of viable cell concentration as described in Equation (1) [37,49,50]. N0 is the viable cell concentration (log CFU/g) before spray drying and N_t_ is the viable cell concentration (log CFU/g) after spray drying. For gastrointestinal simulation, N_0_ is the initial viable cell concentration (log CFU/g) before the simulation, and N_t_ is the viable concentration (log CFU/g) after the simulation.

Log reduction = log N_0_ − log N_t_ (1)

### 2.6. Gastrointestinal Simulation

The survival during GIT simulation was studied to monitor viability loss during consumption. The method was adapted from a previous study with modifications [44]. In general, spray-dried and free cells of *P. acidilactici* (control) were sequentially exposed to simulated gastric juice (SGJ) for 2 h and simulated intestinal juice (SIJ) for 4 h. Firstly, the formulation for SGJ was prepared according to a study by Kulkarni et al. [51]. Briefly, MRS broth were added with glucose (3.5 g/L), NaCl (2.05 g/L), KH_2_PO_4_ (0.60 g/L), CaCl_2_ (0.11 g/L), and KCl (0.37 g/L), and then adjusted to pH 2.0 using 1 M HCl. Then, pepsin (from porcine stomach mucosa, Sigma Chemical Co., St. Louis, MO, USA) was added to the sterile SGJ stock by 13.3 mg/L. Sterile 25-mL Erlenmeyer flasks were prepared and filled with 9 mL of sterile SGJ. Into each 9 mL sterile SGJ solution, 1 g of spray-dried samples or 1 mL of free cells (control) were added, producing suspension that was homogenized using a vortex for 2 min. Viable cell counting was performed to obtain initial cell concentration before GIT simulation. Next, the suspensions were incubated in a shaker incubator (TOU-50N Orbital Shaker Incubator, MRC, Holon, Israel) for 2 h at 37 °C and 150 rpm for gastric juice simulation. Once the incubation in SGJ was done, the samples were neutralised with 1 M NaOH to pH 7, quenching the pepsin enzymatic reaction. Viable cell counting was performed to obtain viable cell concentration after SGJ simulation. Then, porcine bile (0.7% *w*/*v*; Sigma Chemical Co., St. Louis, MO, USA) was added to each flask and incubated at 37 °C and 150 rpm for 4 h for SIJ simulation. Once the incubation was complete, the last viable cell counting was conducted to obtain viable cell concentration after sequential SGJ and SIJ simulation.

### 2.7. Storage Test

*P. acidilactici* viability during storage was investigated to observe the viable cell reduction during storage at room temperature for 4 months. During the 1st month, the viable cell count of spray-dried *P. acidilactici* was measured every week, and then the frequency was changed to once every month. Viable cell counting was done as described in Section 2.3.

### 2.8. Physicochemical Analysis

The production yield was expressed in percentage, calculated using Equation (2). The mass of encapsulation material used in all samples was 200 g. After weighing, the powder was stored in a sealed aluminium foil bag until further analysis.

(2) $$Production\;Yield=\frac{\;Mass\;of\;spray-dried\;powder\;\left(g\right)\times 100\%}{Mass\;of\;encapsulation\;material\;\left(g\right)}$$

The moisture content analysis was adapted from a previous study [52]. A gram of sample was heated at 105 °C for 5 min and the average moisture content was measured using a moisture analyser (Ohaus^®^ MB-45 moisture balances, Parsippany, NJ, USA). The water activity (a_w_) was determined by Pawkit Water Activity Meter (Decagon, Pullman, WA, USA). Samples were poured into the water activity measurement cup, and the water activity meter analysed the moisture content for 5 min [52,53].

### 2.9. Data Analysis

All the experiments were performed in triplicates. Data from the experiment were analysed by GraphPad Prism version 8.0.0 for Windows, GraphPad Software, San Diego. Ca, USA, www.graphad.com, accessed on 19 November 2022. The effects were considered significant at *p* < 0.05. Results were presented as the mean ± standard deviation. Statistical analysis on encapsulation efficiency and physicochemical properties (excluding FT-IR and powder morphology) was performed by one-way analysis of variance (ANOVA). Meanwhile, statistical analysis on gastrointestinal simulation and storage test was performed using a two-way analysis of variance (ANOVA). Tukey’s honestly significant difference test (*p* < 0.05) was conducted as a posteriori contrast after rejecting the null hypothesis.

## 3. Results and Discussion

### 3.1. Survivability of Spray-Dried P. acidilactici

Spray drying is mainly utilized to transform liquid into powder for extended shelf-life and ease of storage and transportation, and, compared to freeze drying, it is considered a low-cost process [32,33,34]. However, when it is used for probiotic encapsulation, spray drying has been associated with reduced cell viability as the inlet air temperature increases [54,55]. Such a viability loss is related to high heat stress resulting from drying, which may damage the structural integrity of cellular components and membranes [54,55,56]. This study investigates *P. acidilactici* viability after spray drying in an equal ratio of WPI:GA at different inlet air temperatures (120 °C, 150 °C, and 170 °C).

This study found that there was no significant difference (*p* > 0.05) in viability loss when the inlet temperature was increased from 120 °C to 150 °C (Table 1). S120 and S150 had 0.59 and 0.62 log cycle reductions, respectively. Meanwhile, the viability decreased significantly (*p* < 0.05) by 1.36 log cycles, when the temperature was increased to 170 °C. Viable cell loss induced by high inlet air temperature has also been reported in other studies [28,29,31,57]. Behboudi-Jobbehdar et al. [57] tested different inlet air temperatures (120 °C, 140 °C and 160 °C) and reported that the viability of *Lactobacillus acidophilus* spray-dried in a combination of maltodextrin, whey protein, and glucose decreased from 9.02 to 7.37 log CFU/g as the inlet temperature increased. A similar pattern was reported for *L. acidophilus* encapsulated in WPI-GA spray-dried at inlet temperatures of 100 °C, 120 °C and 140 °C [44].

In addition to inlet temperature, selecting the appropriate encapsulation material also plays a critical role in protecting the probiotics during spray drying, storage, and GIT transit [21,28,29]. GA is one of the most widely studied probiotic encapsulating agents. Its protective effect has been observed during spray drying of multiple probiotic species, including *L. acidophilus* [39,44], *Enterococcus faecalis* [58], *Saccharomyces cerevisiae* var. *boulardii* [47], and *Lactobacillus plantarum* [59]. WPI is another commonly used probiotic encapsulating material known for its high resistance and stability against pepsin digestion, providing better protection against stomach conditions. To enhance its protective effect, GA and WPI are usually used together [44,59,60]. When combined, WPI and GA can create an emulsion that protects *P. acidilactici* from cellular injury during spray drying [60,61]. The resulting emulsion demonstrates suitable physicochemical properties, such as molecular weight, glass transition, crystallinity, diffusibility, film-forming properties, and feed solution viscosity, that are suitable for spray drying encapsulation [43,46,62]. Since the success of encapsulation depends on the WPI-GA ratio [44,59,60], this study tested different WPI-GA ratios (1:3, 1:1, and 3:1) using fixed inlet air temperature (150 °C).

As shown in Table 2, all WPI:GA ratios generated *P. acidilactici* powder with high viable cell counts (7.49–7.78 log CFU/g), and no significant difference (*p* > 0.05) in viability loss (0.58–1.11 log cycles) was observed between samples. Past studies had shown different success when WPI and GA were used for encapsulating probiotics. For example, a viable cell reduction of 1.43 log CFU/g was achieved by Leylak et al. [44] using a 1:0 WPI:GA ratio and inlet temperature of 120 °C. Meanwhile, Sharifi et al. [59] reported a higher log reduction of approximately 3 log CFU/g in *L. plantarum* spray-dried using WPI-GA alone or in combination with phytosterol. Similarly, Eratte et al. [60] reported about 5.4 and 4.1 log cycle loss of *Lactobacillus casei* spray-dried using WPI-GA alone and in combination with omega 3, respectively.

### 3.2. Production Yield, Moisture Content, and Water Activity of Spray-Dried P. acidilactici

Increasing the inlet air temperature was found to increase the spray-dried *P. acidilactici* yield (Table 3), which was consistent with the previous studies [63,64,65]. The lowest production yield was obtained with inlet air temperature 120 °C (58.97%), followed by 150 °C (63.12%) and 170 °C (69.62%). Since higher inlet air temperature is associated with a greater heat transfer efficiency, the probability of carrier material sticking onto the drying chamber wall is reduced, therefore increasing the production yield [66,67]. Increasing inlet air temperature was also found to reduce the powder’s moisture content and water activity (a_w_) (Table 2), which are highly desirable for a longer shelf-life [31,68,69,70]. As shown in Table 3, all spray-dried *P. acidilactici* had moisture content within a range of 4.66–6.80%, which was comparable to other studies [63,64,65] and considered suitable for storage at room temperature (25 °C) [71,72,73]. Furthermore, the a_w_ of all spray-dried *P. acidilactici* fell within the range of 0.16–0.29, which is sufficient for preventing bacteria from being metabolically active until consumption [69]. According to the literature, probiotic powders are recommended to have a_w_ value of around 0.25 to preserve its viability and prevent undesirable microbial growth [68,69,74].

The production yield of all WPI-GA ratios ranges from 63.12 to 67.10%, and no significant differences (*p* > 0.05) were observed between samples (Table 4). The moisture content (4.66–5.91%) also fell within the acceptable range for room temperature (25 °C) storage with minimum viability loss [71,72,73]. The moisture content was found to increase with increasing GA proportion, which could be attributed to the water holding capacity of proteins (1.5–2.6%) contained in the GA [75,76]. Regardless, the a_w_ between all samples showed no significant difference (*p* > 0.05), ranging from 0.17 to 0.21.

### 3.3. Survival of P. acidilactici in Simulated GIT

Encapsulation is expected to retain the probiotic viability during gastrointestinal digestion [44,56,62]. In this study, *P. acidilactici* spray-dried with various inlet air temperatures and WPI:GA ratios was subjected to a survivability test in simulated GIT, comprising sequential exposure to SGJ and SIJ. As shown in Table 5, no viable cells were detected in the non-spray-dried *P. acidilactici* after 2 h of incubation in SGJ (reduction of 8 log CFU/g). By the end of the simulation, S150 exhibited the lowest log reduction (0.50 log CFU/g), followed by S120 (0.77 log CFU/g) and S170 (0.78 log CFU/g). According to the literature, to a certain extent, a higher inlet air temperature could improve the survivability of spray-dried probiotics under all pH conditions [47,56,62]. Studies by Arslan et al. and Baghwat et al. [47,62] suggested that higher inlet temperature could affect the powder’s physical form by forming an impermeable and solid structure, providing better protection to the probiotics. Meanwhile, increasing the inlet temperature further could generate incomplete coverage, as previously observed by Ng et al. [77], decreasing the probiotics’ survivability in adverse conditions.

The viable cell concentration of *P. acidilactici* encapsulated in different WPI-GA ratios during GIT simulation is shown in Table 6. All WPI-GA ratios maintained 5.62–6.41 log CFU/g of viable cells after 2 h incubation in SGJ. The viable cell reduction of 0.3–0.75 log CFU/g observed might have been caused by the porous nature of the encapsulation wall and possible WPI digestion by pepsin, allowing some acid to penetrate the matrix and cause cell injuries [62,78]. Higher WPI content in WG31 might have been able to withstand this phenomenon, thus retaining a high number of viable cells within the encapsulation matrix. Another possible contributing factor is the surface topography of the encapsulating matrix, as suggested by Doherty et al. [79]. The disrupted surface topography (such as dents and wrinkled surface) could increase the surface area of the encapsulation wall [79], which corresponds to higher powder diffusion and active components (the probiotic cell) release rate [80,81]. The encapsulation wall with low WPI content (WG13) might have experienced such a disrupted surface topography, verified by performing scanning electron microscopy (SEM) as described in Section 3.6.

After 4 h of incubation in SIJ, WG31 maintained stable viability as no significant change (*p* > 0.05) was observed from the initial counts. On the other hand, viable cell counts in WG11 and WG13 decreased significantly (*p* < 0.05) by 0.5 and 0.75 log cycles, respectively, by the end of the simulation. Bile is detrimental to bacteria, possibly affecting cell permeability and interactions between the membrane and its environment [62,82]. It disrupts the cell membrane by altering the lipid matrix in the cell membrane due to its detergent and lipophilic properties [83]. The bile extract also contains pancreatin and lipase, which are potentially harmful to bacteria [84]. The results of this study suggest that reducing the WPI:GA ratio to 1:3 (WG13) could not provide sufficient protection against bile extract in simulated SIJ. The inability of WG13 to withstand acid and pepsin digestion during the SGJ simulation could have promoted the diffusion of bile extract through the encapsulation wall during simulated SIJ.

### 3.4. Survival of Spray-Dried P. acidilactici during Storage

Storage testing is an essential evaluation to determine the product shelf life and ensure overall product quality over time. The International Dairy Federation and previous reports [1,8,9,10] suggest a minimum probiotic cell concentration of 7 log CFU/g should be retained until the end of the shelf life to maintain its product efficacy. Since drying causes damage to the cell membrane, proteins, and nucleic acids, preserving probiotics viability in the powder form over a long period of storage at ambient temperature remains a challenge. In this study, the viable cell counts of spray-dried *P. acidilactici* were evaluated over four months of storage at room temperature (25 °C). During the 1st month, the viability was measured weekly, and then the measurement was performed monthly.

**Figure 1:**
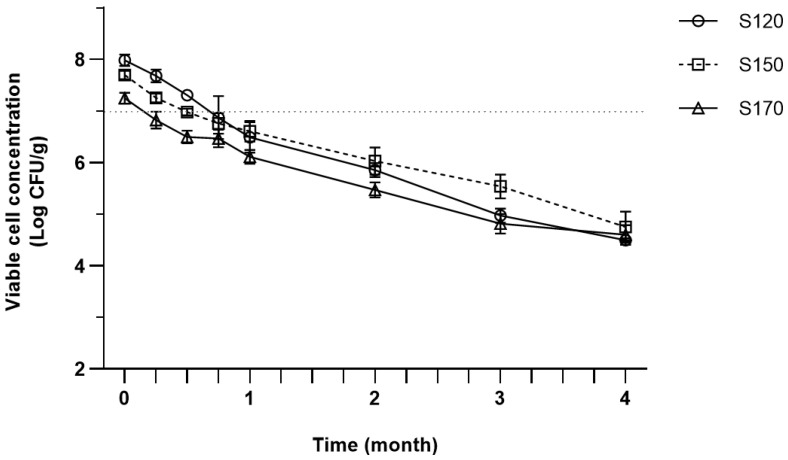
Viable cell concentration of spray-dried *P. acidilactici* produced by different inlet air temperatures during storage at room temperature (25 °C). Error bars represent ± Standard Error of the Mean (SEM) of three replicates (*n* = 3). Dotted lines in y axis at 7 log CFU/g shows the suggested minimum concentration of viable probiotics that should be incorporated in the final product to exert its optimum health benefits.

The survival of *P. acidilactici* spray-dried at different inlet air temperature over 4 months of storage is presented in Figure 1. As depicted in Figure 1, the viability of *P. acidilactici* gradually decreased over time from 7.98–7.25 log CFU/g to 4.5–4.75 log CFU/g by the end of storage. A similar declining pattern was also observed in the previous studies [29,44,85]. Viable cell counts reached below 7 log CFU/g more rapidly with the increasing inlet temperature, which correlates with lower initial counts in higher inlet temperature.

**Figure 2:**
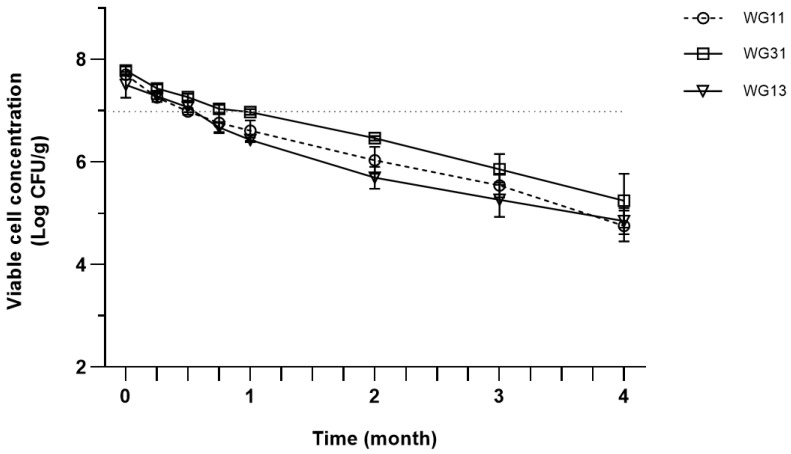
Viable cell concentration of spray-dried *P. acidilactici* produced in different ratios of encapsulation matrices during storage at room temperature (25 °C). Error bars represent ± standard error of the mean (SEM) of three replicates (*n* = 3).

All samples with different WPI:GA ratios also follow a similar declining trend during four months of storage (Figure 2). However, all samples maintained high viability above 7 log CFU/g until the 3rd week of storage, then continuously decreased to 4.75–5.24 log CFU/g by the end of the storage test.

The survival of probiotics powder during storage is associated with the storage temperature, and a_w_. Abe et al. [86] found that the viability of *B. longum* powder increases with decreasing storage temperature and a_w_, while high survivability at 25 °C could be achieved with a_w_ < 0.16. These findings suggest that the a_w_ of the spray-dried *P. acidilactici* obtained in this study might not be low enough to prevent viability loss during storage at 25 °C. In addition, high survivability could be achieved by increasing the powder’s initial viable cell counts in the powder as well as keeping the powder at a lower storage temperature (4–10 °C) [29,44,85,87].

### 3.5. FT-IR Analysis of Spray-Dried P. acidilactici

**Figure 3:**
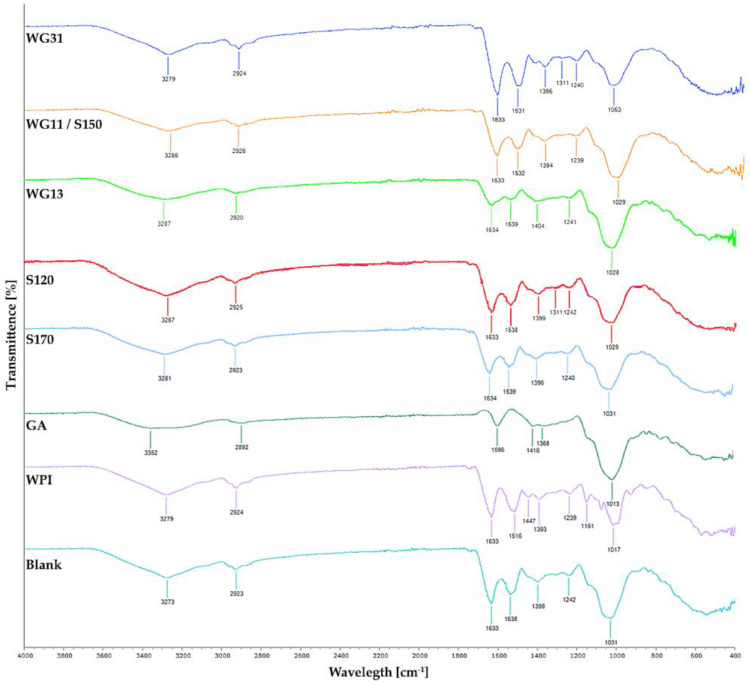
FTIR spectra of encapsulation matrices and spray-dried *P. acidilactici* that were produced in the different inlet air temperature and ratio of encapsulation matrices. WPI: raw Whey Protein Isolate, GA: raw Gum Arabic, Blank: spray-dried WPI:GA at 1:1 ratio.

The FT-IR spectra of raw WPI and GA powder are illustrated in Figure 3. The FT-IR spectra of raw WPI powder (WPI) showed two weak peaks, indicated by high transmittance at 80–90% in the functional group region. The peak around 3260–3280 cm^−1^ is attributable to the amine N-H stretching [59] and 2920–2930 cm^−1^ to the alkyl C-H stretch. The raw GA powder (GA) spectra have two peaks at the functional region with slightly lower transmittance and a broader peak than the WPI profile. The peak within 3500–3000 cm^−1^ may be attributed to the O-H stretching of alcohol/phenol, and the peak around 2892 cm^−1^ is closely related to carboxylic groups. The result of the WPI profile is consistent with the previous study by Bhagwat et al. [62], Sharifi et al. [59], and Rajam and Anandharamakrishnan [36], whereas GA is consistent with studies by Sharifi et al. [59], Santos et al. [88], and Daoub et al. [89].

Apart from the raw encapsulation powder profile, raw WPI:GA (1:1) solution (blank) was spray-dried to identify any structural changes between the biopolymers and to check whether it is retained after spray drying. The blank spectra show WPI dominance in its functional region, while the fingerprint region indicates WPI dominance in 1700–1250 cm^−1^ and GA dominance in the 1250–900 cm^−1^ region. This result suggests no interaction between the wall material and the WPI-GA compound is retained after spray drying.

The FT-IR spectra of *P. acidilactici* spray-dried with different inlet air temperatures are also presented in Figure 3. It is shown that the FT-IR spectra follow a similar profile observed in blank and other WPI-GA combinations reported in the previous study [59], indicating no interaction between wall material and probiotics. Bonds mentioned previously could be seen in all samples which indicate that different inlet air temperature has no impact on the chemical structure of encapsulation materials as well. Similar profiles were also found in the FT-IR spectra of spray-dried *P. acidilactici* samples containing different WPI:GA ratios (Figure 3). A slight change in peak widths in the functional region (around 3500–3000 cm^−1^) corresponds to the increasing GA ratios. This result confirmed that WPI and GA structures were retained after spray drying. Additionally, there is no chemical interaction between the cells and the encapsulation material, comparing the blank and spray-dried *P. acidilactici* sample.

### 3.6. SEM Analysis

**Figure 4:**
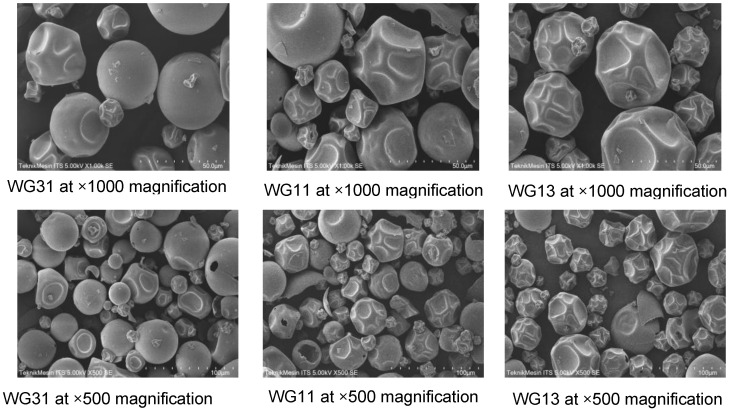
SEM results of spray-dried *P. acidilactici* in different WPI:GA ratios.

The morphology of spray-dried *P. acidilactici* was examined using SEM as shown in Figure 4. Mechanical defects such as fractures, cracks, or holes are unfavourable as they could expose the bacteria to oxidation and lead to viable loss [90]. Surface morphology is often found to be affected by the drying condition, the composition of the feed solution, and the total solid content used in spray drying [91,92]. In this study, the morphology was analysed for the *P. acidilactici* spray-dried at the inlet temperature of 150 °C varying in WPI:GA ratios, since 150 °C could produce a powder with high production yield and good physicochemical properties while retaining high viable cell count.

As depicted in Figure 4, all samples were able to form separated microcapsules without any aggregation between particles. The spray-dried powder mainly contains larger particle sizes between 20–50 μm. WG31 shows a smooth spherical surface, which could be due to the difference in surface tension and viscosity of the final feed solution. In a previous study, WPI was reported to have a lower surface tension than GA [46]. In addition, when WPI and GA were mixed, a higher WPI ratio promoted lower surface tension in the final suspension. This surface tension is closely related to viscosity, a key factor affecting the particle morphology in spray drying [91]. Therefore, the lower viscosity in the WG31, resulting from its lower surface tension, may contribute to its smooth morphological feature.

On the contrary, more dents, wrinkled surfaces, and blow-holes were present in samples WG13 and WG11, potentially due to the increased viscosity of the feed solution with the increasing concentration of GA [93]. During drying, increased viscosity lowers the diffusion rate of water to the surface allowing solid crust formation while some moisture is still trapped inside. Upon further drying the vapor forced its way out by creating holes in the crust. These findings suggest that equal or higher GA ratios were not favourable as they produce wrinkled surface encapsulation, blow-holes, and shredded particles. This suggests that the addition of GA in a low ratio helps WPI withstand the mechanical stress during spray drying.

## 4. Conclusions

This study investigates the effect of inlet air temperature (120 °C, 150 °C and 170 °C) and WPI:GA ratio (1:1, 3:1, 1:3) on spray-dried *P. acidilactici* survivability and physicochemical properties. The results show that increasing the inlet air temperature was favourable in terms of production yield, moisture content, and a_w_. However, increasing the inlet temperature to 170 °C could lead to a significant viability loss. Unlike the inlet air temperature, the effect of WPI:GA ratio on the survival of spray-dried cells, yield, moisture content, and a_w_ was not apparent. Furthermore, increasing the inlet air temperature to 150 °C resulted in the lowest viable cell reduction (0.5 log cycle) after the GIT simulation. For samples containing various WPI:GA concentrations, the viable cell reduction after simulated GIT was significantly lower when a higher WPI proportion (WG11 and WG31) was used. According to the SEM analysis, samples with higher WPI content exhibited a smoother spherical surface, while those containing a higher GA content demonstrated more dents, wrinkled surfaces, and blow-holes. During storage at 25 °C, viable cell counts decreased to below 7 log CFU/g more rapidly as the inlet temperature increased, which correlates with the encapsulation efficiency. Meanwhile, varying WPI:GA ratios did not influence the survivability of spray-dried *P. acidilactici* during storage. FT-IR spectra indicated no chemical interaction between wall materials and *P. acidilactici*. This study confirms that selecting the appropriate inlet air temperature and wall material ratio is vital in obtaining *P. acidilactici* powder with good physicochemical properties, high encapsulation efficiency, and survivability against gastrointestinal and storage conditions. Future investigations could focus on the effect of spray drying parameters on *P. acidilactici* functionalities.

## Figures and Tables

**Table 1:** Survivability of spray-dried *P. acidilactici* produced by different inlet air temperatures.

Sample (Inlet Temperature)	Viable Cell Concentration (Log CFU/g)		Viability Loss (Log Reduction)
	Before Spray Drying	After Spray Drying	
S120 (120 °C)	8.57 ± 0.15 ^aA^	7.98 ± 0.19 ^aB^	0.59 ± 0.32 ^A^
S150 (150 °C)	8.32 ± 0.06 ^aA^	7.69 ± 0.14 ^bA^	0.62 ± 0.09 ^A^
S170 (170 °C)	8.61 ± 0.07 ^aA^	7.25 ± 0.19 ^bB^	1.36 ± 0.24 ^B^

All data represent the mean (*n* = 3) ± standard deviation (SD). Different superscript uppercase letters in the same column (A,B) indicated significant differences. Different superscript lowercase letters in the same row (a,b) indicated significant differences. Statistical analyses were performed using ANOVA Tukey’s multiple comparison test at *p* < 0.05.

**Table 2:** Survivability of spray-dried *P. acidilactici* produced by different WPI-GA ratios.

Sample (WPI:GA)	Viable Cell Concentration (Log CFU/g)		Viability Loss (Log Reduction)
	Before Spray Drying	After Spray Drying	
WG11 (1:1)	8.32 ± 0.06 ^aA^	7.69 ± 0.14 ^bA^	0.62 ± 0.09 ^A^
WG31 (3:1)	8.36 ± 0.32 ^aA^	7.78 ± 0.13 ^bA^	0.58 ± 0.23 ^A^
WG13 (1:3)	8.60 ± 0.04 ^aA^	7.49 ± 0.43 ^bA^	1.11 ± 0.47 ^A^

All data represent the mean (*n* = 3) ± standard deviation (SD). Means sharing superscript uppercase letter (A) in the same column do not differ significantly. Different superscript lowercase letters in the same row (a,b) indicated significant differences. Statistical analyses were performed using ANOVA Tukey’s multiple comparison test at *p* < 0.05.

**Table 3:** Physicochemical properties of spray-dried *P. acidilactici* produced by different inlet air temperatures.

Sample	Inlet Air Temperature (°C)	Production Yield (%)	Moisture Content (%)	Water Activity (a_w_)
S120	120	58.97 ± 0.03 ^b^	6.80 ± 0.92 ^a^	0.29 ± 0.04 ^a^
S150	150	63.12 ± 0.04 ^ab^	5.71 ± 0.28 ^b^	0.21 ± 0.02 ^b^
S170	170	69.62 ± 0.02 ^a^	4.75 ± 0.14 ^c^	0.16 ± 0.02 ^b^

All physicochemical results are represented by the mean (*n* = 3) ± standard deviation of replicated biological samples. Significant differences (*p* < 0.05) via ANOVA and Tukey’s post hoc test are shown by different superscripts (a–c) in the same column.

**Table 4:** Physicochemical properties of spray-dried *P. acidilactici* produced in different WPI-GA ratios.

Sample	WPI:GA Ratio	Production Yield (%)	Moisture Content (%)	Water Activity (a_w_)
WG11	1:1	63.12 ± 4.13 ^a^	5.71 ± 0.29 ^a^	0.21 ± 0.04 ^a^
WG31	3:1	67.10 ± 2.00 ^a^	4.66 ± 0.48 ^b^	0.19 ± 0.02 ^a^
WG13	1:3	65.97 ± 4.06 ^a^	5.91 ± 0.42 ^a^	0.17 ± 0.02 ^a^

All physicochemical results are represented by the mean (*n* = 3) ± standard deviation of replicated biological samples. Significant differences (*p* < 0.05) via ANOVA and Tukey’s post hoc test are shown by different superscripts (a,b) in the same column.

**Table 5:** Viability of *P. acidilactici* spray-dried at different inlet air temperatures during GIT simulation.

Sample	Viable Cell Concentration (Log CFU/g)			Viability Loss after GIT Simulation (Log Reduction)
	Initial	After SGJ	After SIJ	
Free cell	8.00 ± 0.04 ^aA^	0.00 ± 0.00 ^bA^	0.00 ± 0.00 ^bA^	8.00 ± 0.04 ^A^
S120	7.52 ± 0.14 ^aB^	6.49 ± 0.11 ^bB^	6.75 ± 0.05 ^cB^	0.77 ± 0.17 ^B^
S150	6.60 ± 0.21 ^aC^	6.09 ± 0.21 ^bC^	6.10 ± 0.08 ^bC^	0.50 ± 0.19 ^B^
S170	6.43 ± 0.07 ^aC^	5.59 ± 0.16 ^bD^	5.65 ± 0.07 ^bD^	0.78 ± 0.14 ^B^

All data represent the mean (*n* = 3) ± standard deviation (SD). SGJ: simulated gastric juice; SIJ: simulated intestinal juice that is done sequentially after SGJ. Different superscript uppercase letters in the same column (A–D) indicated significant differences. Different superscript lowercase letters in the same row (a–c) indicated significant differences. Statistical analyses were performed using ANOVA Tukey’s multiple comparison test at *p* < 0.05.

**Table 6:** Viability of *P. acidilactici* spray-dried in different WPI-GA ratios during GIT simulation.

Sample	Viable Cell Concentration (Log CFU/g)			Viability Loss after GIT Simulation (Log Reduction)
	Initial	After SGJ	After SIJ	
Free cell	8.00 ± 0.04 ^aA^	0.00 ± 0.00 ^bA^	0.00 ± 0.00 ^bA^	8.00 ± 0.04 ^A^
WG11	6.60 ± 0.21 ^aB^	6.09 ± 0.21 ^bBC^	6.10 ± 0.08 ^bB^	0.50 ± 0.19 ^BC^
WG31	6.85 ± 0.08 ^aBC^	6.41 ± 0.24 ^bBC^	6.55 ± 0.28 ^abC^	0.30 ± 0.21 ^C^
WG13	5.99 ± 0.12 ^aD^	5.62 ± 0.11 ^bD^	5.24 ± 0.09 ^cD^	0.75 ± 0.04 ^B^

All data represent the mean (*n* = 3) ± standard deviation (SD). SGJ: simulated gastric juice; SIJ: simulated intestinal juice that is done sequentially after SGJ. Different superscript uppercase letters in the same column (A–D) indicated significant differences. Different superscript lowercase letters in the same row (a–c) indicated significant differences. Statistical analyses were performed using ANOVA Tukey’s multiple comparison test at *p* < 0.05.
